# Effects of Sound on Postural Stability during Quiet Standing

**DOI:** 10.1186/1743-0003-8-67

**Published:** 2011-12-15

**Authors:** Sung Ha Park, Kichol Lee, Thurmon Lockhart, Sukwon Kim

**Affiliations:** 1 Department of Industrial and Management Engineering, Hannam University, Daejeon, South Korea; 2 Department of Industrial and Systems Engineering, Virginia Tech, Blacksburg, Virginia, USA; 3 Department of Physical Education, Chonbuk National University, Jeonju-City, South Korea

**Keywords:** Noise, Sound Levels, Frequency, Stability, De Boer's rating score

## Abstract

Loss of postural stability can increase the likelihood of slips and falls in workplaces. The present study intended to extend understanding of the effects of frequency and pressure level of sound on postural stability during standing. Eleven male subjects participated. Standing on a force platform, the subjects' center of pressures were measured under different combinations of pressure level and frequency of the sound. Variables such as the position variability of COP and the length of postural sway path in anterior-posterior (AP) and medio-lateral (ML) direction were evaluated. Subjective ratings of perceived disturbance at each experimental condition were also obtained using a 7-point rating scale. Results showed that the length of sway path and the position variability of COP increased as the frequency of sound increased in posterior-anterior axis. The effect of sound pressure level, however, was not significant on both the postural sway length and the position variability of COP. These results suggested substantial disturbance of standing balance system among subjects exposed to high frequency noise. The results implied that physical workers should be alerted that their abilities of postural balance could be degraded significantly as disturbance caused by a sound existed.

## Background

Falls were often considered to be a leading cause of death at workplace worldwide. According to Ministry of Labor (2002) in South Korea, 426 deaths occurred at the workplace in 2001 due to falls [1]. This placed falls the second leading cause of death injury in 2001 in South Korea [1]. In US, falls were identified for the second most frequent fatal events only to Highway accidents [2]. In 2006, twenty percent of nonfatal cases involving days away from work was caused by fall related incidents (234,450 of 1,183,500 injuries and illnesses) [3]. On average, injuries or illnesses caused by fall-related incidents resulted in 10 days away from work [3]. The annual direct cost from occupational injuries due to slips, trips and falls in US was estimated to exceed $6 billion [4]. And, floors and walkways or ground surfaces were identified for the major sources of fall accidents, 86% of all fall-related injuries.

During standing, postural balance is kept intact by a continuous effort of the musculoskeletal, visual, proprioceptive, or vestibular systems. Postural instability (i.e. a loss of balance), frequently evaluated by a measure in the center of pressure (COP), is directly related to risk of falling [5-8]. The likelihood of postural instability while working can be influenced by environmental, task-related, or personal factors at workplace [9-12]. And, the chance of injuries due to loss of balance potentially increase if one or more among the musculoskeletal, visual, proprioceptive, or vestibular systems are interfered by these factors. For instance, one major function of the vestibular system is to continuously monitor and maintain the postural stability [13,14]. Normal sounds can disturb the postural steadiness because of acute oculomotor responses that may increase postural sway [15]. Considering the relevance of sensory system of the inner ear vestibular organs and organ of Corti, sound should affect the human postural stability [12,13,15-17]. However, postural instability caused by vestibular interferences due to noise has not received adequate attentions from scientific groups in comparison to musculoskeletal interferences.

In occupational environments, workers are exposed to sounds with sufficient intensity (e.g., building and construction, manufacturing). Sounds can affect human postural stability because of relationship between vestibular system and organs of Corti in inner ear. The perceived magnitude of sound is known as the loudness, which is a function of both intensity and frequency. Thus, both the frequency and pressure level can contribute to the postural disturbance. Measurement of postural sway is a simple and common method for assessing postural stability during standing [6,14]. COP displacement has been widely used to make inferences when evaluating neurologic and biomechanics mechanisms of postural control [6]. A study [18] reported that postural instability during standing was decreased in the elderly due to physiological deterioration of vestibular function. Thereafter, a limited amount of research that involved sound has been performed to explain the standing balance system. Juntunen et al. [19] studied the effect of high-energy impulse noise on postural body sway. In this study, they reported that subjects with severe noise-induced hearing loss showed significantly more body sway than healthy controls. In the same study, subjects with more severe hearing loss also showed more postural sway than those with less severe hearing loss. It was not clear, however, whether pressure level or frequency of the sound tended to affect standing balance system for those without hearing loss.

The purpose of this study was to assess the effects of frequency and pressure level of sound on postural stability during standing. The relationship between the postural stability and subjective ratings of perceived disturbance was also of primary concern. The study hypothesized that increased sound frequency and pressure level would deteriorate standing balance system leading to postural instability.

## Subjects and Methods

### Participants

Eleven (11) healthy male subjects were recruited from the student population at Hannam University, Daejeon, South Korea. The Institute Review Board of Hannam University approved the study and informed consent was obtained from all participants before any data collection. They were compensated for her/his participation. No one reported any orthopedic or neurological disorders within the past 12 months. Participants had average stature of 175 cm (SD = 5.7) and average age of 22 (SD = 3.7). All participants were examined by otolaryngologist. The examiner indicated that none of the participants had history of vestibular or/and auditory illnesses. All participants had normal hearing.

### Apparatus

**Figure 1:**
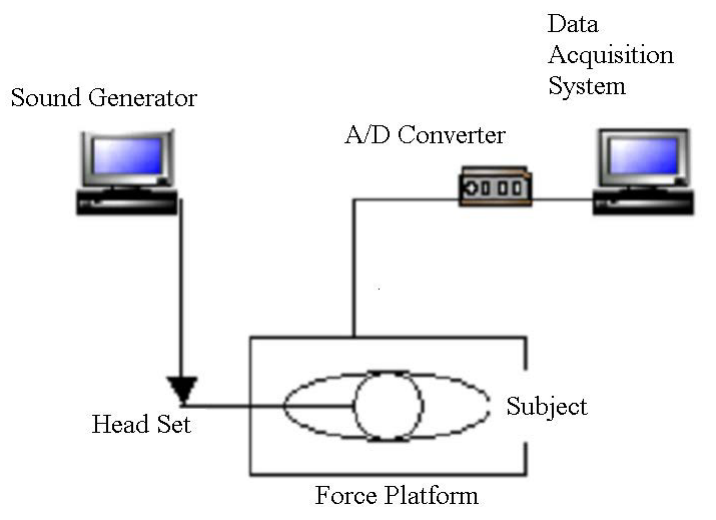


The equipment used for this experiment included a force platform (60 cm × 40 cm × 8.8 cm, Model # K90701, Type 4060-08, BERTEC), a sound level meter (CEL-254, CEL Instruments Ltd, Hitchin, England), and a headphone (JB-M66, jWIN^®^) (Figure 1). Postural reactions were measured using the force plate with sampling rate of 60 Hz. From these data, the position variability of center of pressure (COP) and the length of postural sway path in anterior-posterior (AP) and medio-lateral (ML) direction [11,14] were computed.

In order to produce the various levels of sound pressure and frequency, Sound Generator (http://delphiforfun.org/Programs/soundgen.htm) was utilized. The sound level meter was used to measure the levels of sound (dB). A single tone, produced at different levels of sound pressure and frequency, was continuously exposed to subjects through the headphone during each trial.

### Experimental Design

The study used a repeated-measures experimental design with three levels of sound pressure (45, 90, and 120 dB) and four levels of sound frequency (1000, 2000, 3000, and 4000 Hz). The sound level of 90 dB was A-weighted sound level for the reference duration of 8 hours. The sound level of 45 dB was the allowed daytime levels in quiet residential area. The sound level of 120 dB was the maximum sound level that could be simulated by the laboratory equipment. The 12 trials for each participant were randomly introduced.

Dependent measures included the position variability of COP and the length of postural sway path in anterior-posterior (AP) and medio-lateral (ML) directions. To evaluate the subjective experience of the combined frequency and intensity of the sound, subjective ratings of perceived disturbance at each experimental condition were collected using a 7-point rating scale with verbal descriptions ranging from '1: Not disturbed' to '7: Extremely disturbed'.

### Procedures

Upon arrival, the participants read and signed an informed consent form and, also, they were given verbal explanations of the study protocol. The participants then were instructed to wear the headphone and stand on the force plate with eyes open, head upright, and arms comfortably at their side at all times. Both ears continuously received the tones for 20 seconds although they were asked to stand still before the tone was sent to their ears. When they seemed to stand still, they were exposed for 20 seconds. Then, the data collection began. They were asked to stand quietly with an angle of 30° between feet and heels 10 cm apart. They were asked to stand as still as possible. Between trials, the participants were allowed to take a 5-minute break. Each participant performed 12 (3 × 4) trials. At the end of each trial, the participants rated their perceived disturbance to the sound. No feedback about sound levels, the position variability of COP, and the length of postural sway path was given during the trial.

### Statistical Method

Repeated measures ANOVA was performed by utilizing the JMP statistical packages (SAS Institute Inc. Cary, NC, USA). All measures were within-subject factors.

## Results

ANOVA results for subjective ratings of perceived disturbance showed statistically significant main effect of sound pressure level (SPL, F(2, 20) = 26.651, p < 0.0001) and Frequency (Hz, F(3, 20) = 14.315, p < 0.0001). Average ratings of perceived disturbance were 1.7 ± 1.4, 3.1 ± 1.5, and 4.8 ± 1.8 for sound pressure levels of 45 dB, 90 dB, and 120 dB, respectively. Subjects rated 2.1 ± 1.5, 3.0 ± 1.9, 3.4 ± 1.9, and 4.3 ± 2.1 for frequency levels of 1000 Hz, 2000 Hz, 3000 Hz, and 4000 Hz, respectively. Overall, the subjective ratings of perceived disturbance significantly increased as the frequency and pressure level of sound increased.

X-Y coordinates of COP position were used to compute both the length of sway path and the position variability of COP in anterior-posterior (AP) and medio-lateral (ML) directions. Each of two dependent measures was analyzed using separate repeated-measures analyses of variance (ANOVAs). Significant effects identified by each ANOVA were further evaluated using Student-Newman-Keuls post-hoc comparisons.

### Length of Sway Path

ANOVA results for the length of sway path in anterior-posterior (AP) direction showed no statistically significant main effect of SPL [F(2, 20) = 1.320; p = 0.2895] and two-way interaction of SPL with Frequency [F(6, 60) = 0.869; p = 0.5233]. Although average length of sway path at 45 dB (mean = 0.287 m) was less than the average length of sway path at 120 dB (mean = 0.291 m), the difference was not statistically different. However, *the main effect of Frequency *was significant, F(3, 30) = 2.969, p = 0.0476. Post-hoc comparisons revealed that 3000 Hz and 4000 Hz levels produced significantly longer length of sway path (means = 0.293 and 0.296 m, respectively) as compared with 2000 Hz (mean = 0.276 m). For 1000 Hz (mean = 0.286 m), however, was not significantly different from any other frequency levels. Figure 1 shows mean length of sway path in AP direction. ANOVA results for the length of sway path in medio-lateral (ML) direction did not reveal any statistically significant main effects or two-way interaction of SPL and Frequency (p > 0.05).

### Position Variability

**Figure 2:**
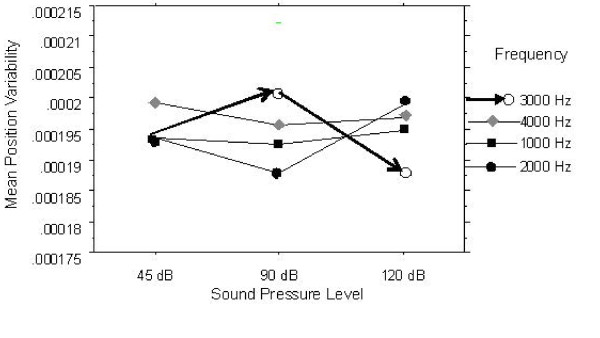


The ANOVA results for position variability of COP showed similar pattern as those for the length of sway path. *The main effect of Frequency *on position variability in *AP direction *was statistically significant, F(3, 30) = 3.043, p = 0.0443. The lowest position variability was produced at frequency of 2000 Hz (mean = 1.776 × 10^-4^) and it was significantly different from 3000 Hz (mean = 1.848 × 10^-4^) and 4000 Hz (mean = 1.861 × 10^-4^). The position variability at 1000 Hz (mean = 1.813 × 10^-4^) was not significantly different from any other frequency levels. The analyses also revealed no statistically significant *main effect of SPL *[F(2, 20) = 0.511; p = 0.6075] and *two-way interaction of SPL × Frequency *[F(6, 60) = 1.674; p = 0.143]. In ML direction, both *main effects *were not significant. The *two-way interaction of SPL with Frequency*, however, was significant, F(6, 60) = 2.274, p = 0.0482. Figure 2 shows two-way interaction plot of sound pressure level and frequency.

## Discussion

The present study was performed to evaluate effects of sound disturbance on human postural stability while standing. The present study found variations in postural sway when subjects were exposed to various levels of sound parameters such as SPL and Frequency. Results revealed that the length of sway path increased as the frequency of sound increased, significantly only in subject's anterior-posterior axis in disagreement with Mainenti et al. [12]. The position variability of center of pressure (COP) changed in the same manner. The position variability and length of sway path were the smallest at frequency level of 2000 Hz and increased below and above this frequency range. The effects of sound pressure level (SPL), however, were not significant on both the postural sway length and the position variability of COP. The duration of exposure to a sound is critical for assessing whether the sound affect one's behavior. A limitation of this study was that the duration of exposure was not included to track the changes in postural stability. The lack of an SPL effect may be due to relatively short duration of exposure (i.e., 20 second) even though SPL was high enough. A study [20] found significant effects of sound disturbances on the postural sway when measured for 51 seconds. In contrast, studies [12,21] indicated no significant effect on the postural sway when the stability was measured shorter durations such as 20 or 30 seconds.

Interestingly, these postural sway parameters showed no clear correlation with the subjective ratings of perceived disturbance. Average ratings of perceived disturbance significantly increased as the frequency and pressure level of sound increased although statistical analysis suggested that, SPL had insignificant effects on postural imbalance. This outcome may suggest that high frequency and/or high sound pressure level would interfere with the vestibular system. But also, another sensory input such as vision [22-24], was utilized to equalize the postural imbalance in the present study. The participants were not asked to close their eyes during the trials. This could alleviate the effects of vestibular inputs on the balance control. Instead, the participants were able to use their visual inputs as an alternative source for balance control. In addition, participants were not asked to perform any tasks except standing still. Hence, they were under very low level of workload - both mental and physical. Adding either or both mental and physical workload could result in different effects because there would be an increase in workload across sensory systems such as visual or somatosensory system. It was suggested by a study [24] that in a well-lit environment with a firm base support, healthy adults depended more on somatory sensory (70%) and vision (10%) information than vestibular (20%) information. However, when an unstable base support was provided, level of dependence on somatory sensory system decreased and more information from vestibular system was used for postural balance [24,25]. This result encourages future experiments allowing participants to stand on surfaces with different slope conditions in order to isolate the effects of vestibular system only on postural stability. Another limitation was lack of pink noise and low frequency for the levels of sound. Since pink noise contains all frequency spectrum and equal energy in each octave bands, the effect of SPL amplitude would be easier to detect when participants were subjected to a pink noise opposed to particular frequencies. Lack of sound levels lower than 1000 Hz was another limitation of this study since human bodies transmit low frequencies better and high amplitude low frequency sounds could vibrate vestibular system more than high frequency sounds.

In conclusion, the present study demonstrated that the magnitudes of postural body sway were different under certain frequency band noises. This suggests substantial disturbance of standing balance system among subjects exposed to excessive sound, mostly at high frequencies. Common sources of noise are power tools, airplanes, chain saws, and many work environments. Physical workers exposed to those work environments should be alerted that their abilities of postural balance diminish significantly.

## Competing interests

The authors declare that they have no competing interests.

## Authors' contributions

SP and SK have made substantial contributions to conception and design, interpretation of data and SP, TL, and SK have been involved in drafting and revising the manuscript. KL has been involved in acquisition of data and analysis of data. All authors read and approved the final manuscript.
